# Tuberculosis Hospitalization Fees and Bed Utilization in China from 1999 to 2009: The Results of a National Survey of Tuberculosis Specialized Hospitals

**DOI:** 10.1371/journal.pone.0139901

**Published:** 2015-10-12

**Authors:** Jian Du, Dirlikov Emilio, Yu Pang, Yan Ma, Fengling Mi, Yuhong Liu, Liang Li

**Affiliations:** 1 Beijing Chest Hospital, Capital Medical University, Beijing, China; 2 Beijing Tuberculosis and Thoracic Tumor Research Institute, Beijing, China; 3 Administration Office, Clinical Center on Tuberculosis Control, China CDC, Beijing, China; 4 Department of Anthropology, McGill University, Montreal, Canada; 5 National Center for Tuberculosis Control and Prevention, Chinese Center for Disease Control and Prevention, Beijing, China; Public Health Agency of Barcelona, SPAIN

## Abstract

**Background:**

China is transitioning towards concentrating tuberculosis (TB) diagnostic and treatment services in hospitals, while the Centers of Disease Control and Prevention (CDC) system will retain important public health functions. Patient expenditure incurred through hospitalization may lead to barriers to TB care or interruption of treatment.

**Methodology/Principal Findings:**

We conducted a national survey of TB specialized hospitals to determine hospitalization fees and hospital bed utilization in 1999, 2004, and 2009. Hospitalization of TB patients increased 185.3% from 1999 to 2009. While the average hospitalization fees also increased, the proportion of those fees in relation to GDP per capita decreased. Hospitalization fees differed across the three regions (eastern, central, and western). Using a least standard difference (LSD) paired analysis, in 2004, the difference in hospitalization fees was significant when comparing eastern and central provinces (*p*<0.001) as well as to western provinces (*p*<0.001). In 2009, the difference remained statistically significant when comparing eastern province hospitalization fees with central provinces (*p*<0.001) and western provinces (*p* = 0.008). In 2004 and 2009, the cost associated with hospitalization as a proportion of GDP per capita was highest in the western region. The average in-patient stay decreased from 33 days in 1999 to 26 and 27 days in 2004 and 2009 respectively. Finally, hospital bed utilization in all three regions increased over this period.

**Conclusions/Significance:**

Our findings show that both the total number of in-patients and hospitalization fees increased from 1999 to 2009, though the proportion of hospitalization fees to GDP per capita decreased. As diagnostic services move to hospitals, regulatory and monitoring mechanisms should be established, and hospitals should make use of the experience garnered by the CDC system through continued strong collaborations. Infrastructure and social protection mechanisms in high burden areas, such as in the western region, should be strengthened.

## Introduction

Tuberculosis (TB) remains a major cause of morbidity and mortality the world over. In 2013, World Health Organization (WHO) estimates that 9.0 million (range, 8.6–9.4 million) incident cases occurred globally, and approximately 1.1 million (range, .98–1.3 million) deaths were attributed to TB, with an additional 360,000 (range, 310,000–410,000) deaths among HIV-positive TB cases [1].

China has made significant headway in TB control. From 1990 to 2010, the prevalence of smear-positive TB fell from 170 cases (range, 166–174) to 59 cases (range, 49–72) per 100,000 population [2]. The results of the 2000 and 2010 national prevalence surveys showed that the prevalence of smear-negative pulmonary TB decreased by 48%, while prevalence of culture-positive patients decreased by 28% [3,4]. Progress has in part been due to the implementation of TB-specific programs, such as strengthened TB-specific infrastructure [5] and health insurance programs [6], including TB-specific publically-funded insurance schemes [7]. Starting in 1991, China scaled-up free diagnostic and treatment services in line with WHO-recommended DOTS strategy (originally an acronym for “directly-observed treatment, short-course”), reaching 100% coverage in 2005 [2,8,9]. As a disease associated with poverty [10,11], China’s economic growth has also impacted the national TB epidemic. According to the World Bank, national GDP per capita increase from US$314.43 in 1990, to US$949.18 in 2000, to US$4,433.34 in 2010; in 2013, it was $6,807.43 [12].

Despite such progress, China remains the country with the second largest burden of both drug-sensitive and drug-resistant TB. In 2013, WHO estimates there were at any given time 1.3 million (range, 1.1–1.5 million) prevalent cases, while 980,000 (range, .91 to 1.1 million) incident cases occurred [1]. An estimated 5.7% (range, 4.5%-7%) of new cases and 25.6% (range, 22%-30%) of retreatment cases are multidrug-resistant (MDR), defined as resistance to both isoniazid (INH) and rifampicin (RMP) [13]. In 2013, MDR-TB among notified new and retreatment cases of pulmonary TB accounted for 45,000 (range 35,000–55,000) and 9,200 (range, 7,800–11,000) cases respectively. That year, only 4,183 cases were laboratory-confirmed, and 2,184 were initiated on treatment [1].

In China, two systems provide clinical services for TB patients: the public health system and the hospital system [5]. The public health system is completely funded by the government, and TB control activities are conducted through the Centers for Disease Control and Prevention (CDCs) system as well as TB dispensaries. Through the public health system, TB patients are provided with a free diagnostic and treatment services. The hospital-based system is comprised of: TB specialized hospitals, infectious disease hospitals, and general hospitals with a TB-specific department. Hospitals are not fully funded by the government, and TB patient diagnostic and clinical services may be partially reimbursed through health insurance schemes.

As part of China’s on-going health reforms, under the 12^th^Five-Year National TB Plan (NTP) for 2011–2015 diagnostic services are being shifted to hospitals. This will simplify TB patient referrals and reduce cases lost to follow-up [14–16]. Under the NTP, the public health system retains important public health functions, including patient follow-up.

As a disease associated with poverty, economic factors play an important role in the emergence of active cases as well as treatment success. In this article, we analyze hospitalization fees associated with TB in-patient treatment over time using data collected through a national survey of TB specialized hospitals. We focus on: the number of in-patients treated, hospitalization fees, and in-patient hospital bed utilization.

## Methods

### Ethics Statement

The protocols applied in this study were approved by the Ethics Committee of the Beijing Chest Hospital, affiliated to Capital Medical University. All surveyed hospital staff provided written informed consent.

### Survey Design

In 2010, the Clinical Center on Tuberculosis (CCTB), China CDC conducted a national survey of TB specialized hospitals to assess hospitalization fees and hospital bed utilization over time. The survey was distributed to all municipal and provincial-level TB hospitals with at least 30 TB-dedicated beds, including TB specialized hospitals, pulmonary hospitals, chest hospitals, infectious disease hospitals, and general hospitals with dedicated TB departments.

The survey was designed by CCTB staff, and surveys were distributed electronically to the appropriate hospital departmental heads. Data were requested for 1999, 2004, and 2009, including: the total number of TB patients treated, hospitalization fees, average length of hospitalization, and hospital bed utilization. Additional data on provincial and regional gross domestic product (GDP) per capita were sourced from CEInet [17].

### Study Definitions

**Fig 1:**
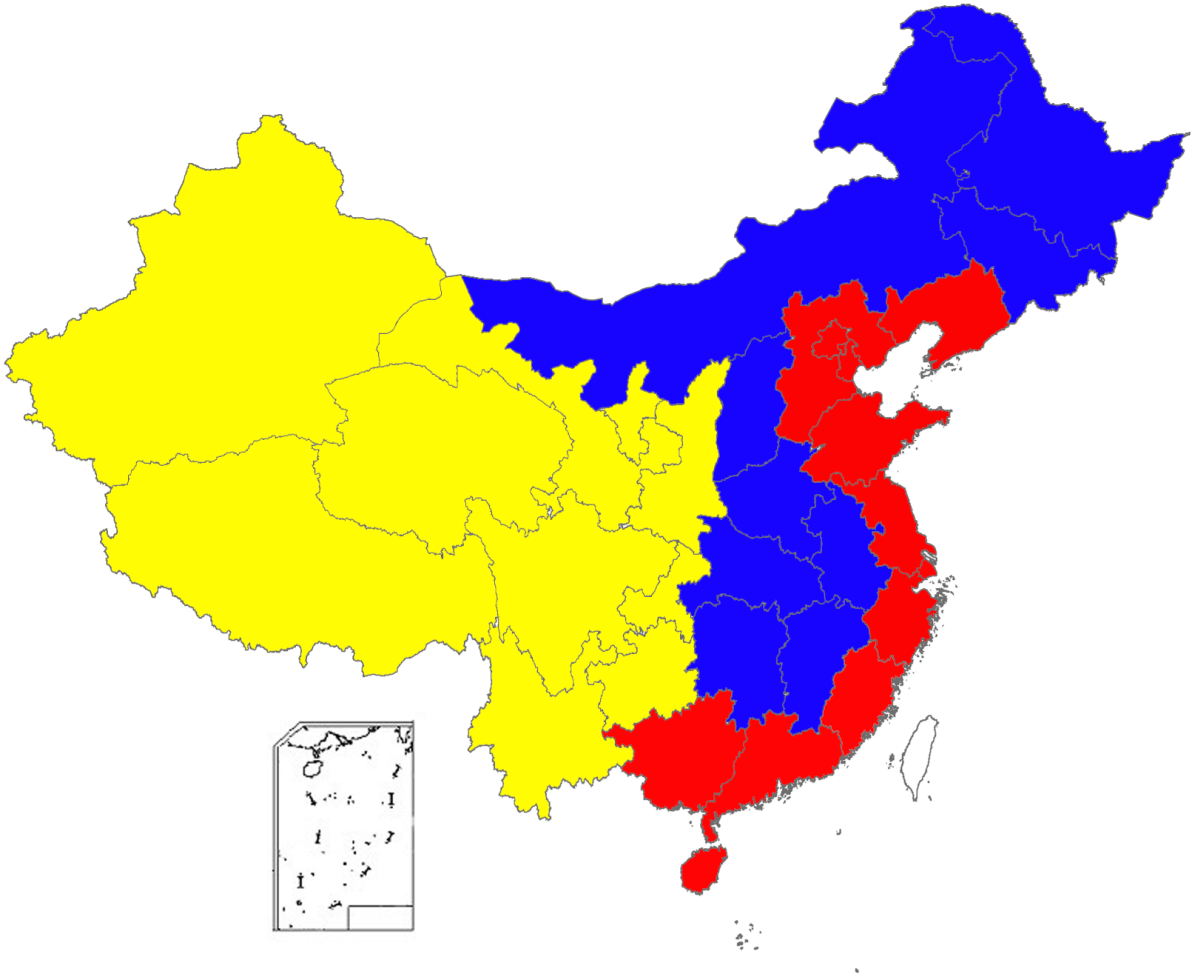
Map of China’s 31 provinces, by region (eastern, central, western). Eastern region provinces (Red): Beijing, Fujian, Guangdong, Guangxi, Hainan, Hebei, Jiangsu, Liaoning, Shandong, Shanghai, Tianjin, and Zhejiang. Central region provinces (Blue): Anhui, Heilongjiang, Henan, Hubei, Hunan, Inner Mongolia, Jiangxi, Jilin, and Shanxi. Western region provinces (Yellow): Chongqing, Gansu, Guizhou, Ningxia, Qinghai, Shaanxi, Sichuan, Xinjiang, Yunnan, and Xizang.

China has three primary administrative levels: provinces, counties, and townships. Municipalities are sub-provincial administrative units, which are further divided into district and county-level administrative units. Further, China’s 31 provincial-level administrative units are grouped into three regions or “economic belts”: eastern, central, and western (Fig 1) [18]. Table 1 provides an overview of regional differentials in terms of TB prevalence, number of TB specialized hospitals, and GDP per capita.

**Table 1:** Comparison of TB Epidemics, TB specialized hospitals, and GDP per capita among different regions of China.

Region	No. of pulmonary TB Patients (millions) (%)^a^	No. of TB Hospitals^b^	GDP per capita in RMB (USD)^c^
**Eastern**	1.734 (29.8)	93 (45.8)	44,732 (~6,548)
**Central**	2.071 (35.6)	84 (41.4)	21,022 (~3,077)
**Western**	2.013 (34.6)	26 (12.8)	19,306 (~2,825)

^a^ As estimated by the fifth national tuberculosis prevalence survey and the sixth nationwide population census, both conducted in 2010 [16].

^b^ As described by Du et al. (2014) [5].

^c^ Data for 2009 gathered from CEInet [17] and converted to USD on x-rates.com using average conversion rates for that year.

In this study, we calculate “hospital bed utilization” as the percentage of hospital beds used by TB in-patients at any given time, as self-reported by surveyed TB specialized hospitals.

### Data analysis

All data were double-entered by two CCTB staff into Epi-Info software (Atlanta, GA). The mean annual percentage change was used to describe change over time. Multiple associations were tested using an ANOVA, whereby values *p*<0.05 were considered statistically significant. Least significant difference (LSD) was used to test bivariate associations. The statistics in this article were generated using SPSS 16.0.

## Results

### Number of in-patients

**Fig 2:**
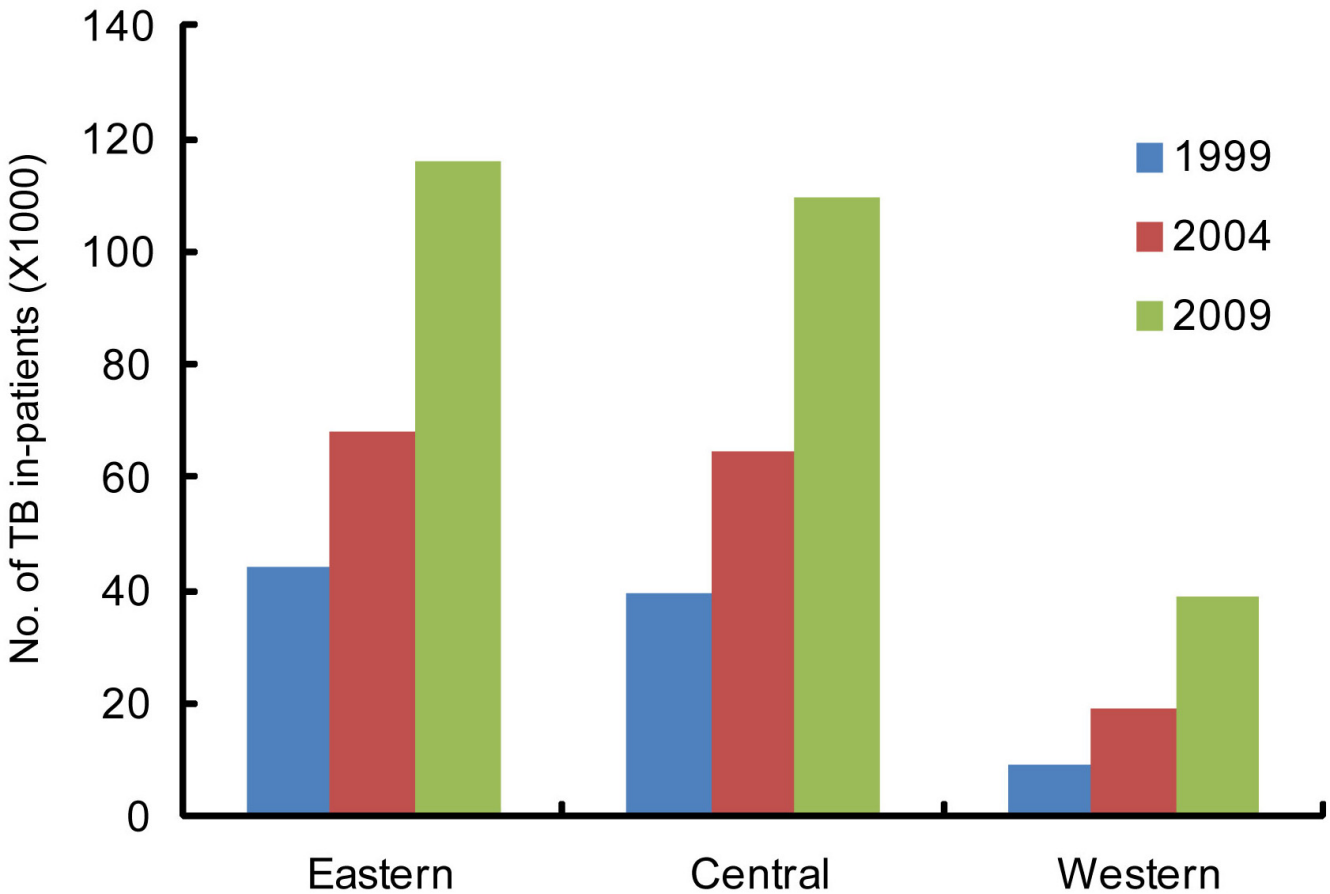
Numbers of patients, per region, per year.

Nationally, the total number of TB in-patients treated at TB specialized hospitals increased from 1999 to 2009 (Fig 2 and S1 Table). In 1999, 92,708 TB in-patients were treated, while in 2004 there were 151,779 in-patients, and in 2009 there were 264,510 in-patients. In comparison to the 1999 baseline, this represents increases of 63.7% and 185.3% for 2004 and 2009, respectively. In each year surveyed, provinces in the eastern region treated the most patients in comparison to central and western provinces. Of patients treated in 2009, 43.7% were in eastern provinces, 41.5% were in central provinces, and 14.8% were in western provinces.

### Hospitalization fees

The average cost associated with hospitalization increased from 1999 to 2004 (Table 2). In 1999, the average cost was 3,389 RMB, which constituted 47.3% of GDP per capita for that year; in 2004, the cost was 4,636 RMB or 37.6% of GDP per capita for that year, and in 2009, the average cost of in-patient hospitalization was 6,631 RMB, or 25.9% of the GDP per capita for that year. In 2004 and 2009, the cost associated with hospitalization as a proportion of GDP per capita was highest in the western region (Table 2 and S2 Table).

**Table 2:** Breakdown of hospitalization fees and GDP per capita, per region.

Region	1999			2004			2009		
	Average Hospitalization Fees	GDP per capita	As a percentage of GDP per capita^a^	Average Hospitalization Fees	GDP per capita	As a percentage of GDP per capita^a^	Average Hospitalization Fees	GDP per capita	As a percentage of GDP per capita^a^
**Eastern**	4266±3787	12700	33.60%	5842±3503	23130	25.30%	7952±4592	44732	17.80%
**Central**	2800±2397	5412	51.70%	3587±2018	9844	36.40%	5469±2724	21022	26.00%
**Western**	2685±1695	4632	58.00%	3436±1941	8462	40.60%	5569±3304	19306	28.80%
**National**	3389±3062	7159	47.30%	4636±3026	12336	37.60%	6631±3944	25605	25.90%

^a^ Average hospitalization fees as a percentage of GDP per capita. Average hospitalization fee and GDP per capita in RMB.

Hospitalization fees differed across the three regions, by year. In 1999, hospitalization fees in eastern provinces differed significantly from central and western provinces (F = 4.24, *p* = 0.016). Using a LSD paired analysis, however, the difference in hospitalization was not significant when comparing eastern provinces to central (*p* = 0.07) or western (*p* = 0.058) provinces. In 2004, the difference in hospitalization fees was significant when comparing eastern and central provinces (*p*<0.001) as well as to western provinces (*p*<0.001); no difference was observed between central and western provinces (*p* = 0.84). In 2009, the difference remained statistically significant when comparing eastern province hospitalization fees with central provinces (*p*<0.001) and western provinces (*p* = 0.008); no difference was observed between central and western provinces (*p* = 0.91).

### Hospital bed utilization

Nationally, the average number of days of hospitalization was 33 days in 1999, 26 days in 2004, and 27 days in 2009 (Table 3). For all three years, the average number of days of hospitalization was greatest in central provinces, which was 36 days in 1999 and 28 days in both 2004 and 2009. Nationally, the percentage of hospital bed utilization increased from 60% in 1999 to 71% in 2004, and 87% in 2009. Hospital bed utilization also increased across all three regions during this period.

**Table 3:** Breakdown of hospital bed usage, per region.

Region	1999			2004			2009		
	Average No. of In-Patient Days	Average Cost per Day of Hospitalization (RMB)	Average Hospital Bed Utilization	Average No. of In-Patient Days	Average Cost per Day of Hospitalization (RMB)	Average Hospital Bed Utilization	Average No. of In-Patient Days	Average Cost per Day of Hospitalization (RMB)	Average Hospital Bed Utilization
**Eastern**	31	138	62%	26	225	75%	26	306	87%
**Central**	36	78	59%	28	128	68%	28	195	87%
**Western**	28	96	59%	21	164	68%	23	242	89%
**National**	33	103	60%	26	178	71%	27	246	87%

## Discussion

As shown in this study, hospitalization of TB patients increased 185.3% from 1999 to 2009. While the average fees associated with hospitalization increased, hospitalization fees as a proportion of GDP per capita decreased. The average in-patient stay decreased from 33 days in 1999 to 26 and 27 days in 2004 and 2009 respectively. Hospital bed utilization in all three regions increased from 1999 to 2009, despite declining national incidence rates over this period. Our findings contribute to research on TB in-patient expenditure in China. A report of TB in-patients at a hospital in Guangdong Province found that the average stay was 27 days in 2004, and the average costs associated with hospitalization were14,865 RMB. The high fees associated to hospitalization may have attributed to the higher proportion of refractory and drug-resistant TB patients in this hospital [19]. In a recent retrospective study in Zhangjiagang of China, the average in-patient cost per TB patient was 6,978.6 RMB [20].

Hospital-based care for TB patients has several advantages, particularly during the initial two-month intensive treatment period. For example, health care workers in TB specialized hospitals are more experienced in treating complicated cases, such as adverse effects from standardized treatment, and may be better suited to provide individualized care. However, hospital-based TB patient care presents several challenges, which will need to be attended to as diagnostic services in China shift to the hospital-based system.

First, as elsewhere, in-patient TB care in China is more expensive than treatment conducted on a strictly out-patient basis [21, 22]. The results of this study show that although there was an increase in fees associated with hospitalization, the cost of hospitalization as a proportion of GPD per capita has decreased. This is in part explained by China’s rapid economic growth. Additionally, the application of new diagnostic tools and treatment regimens since 1999 may have contributed to more accurate and timely diagnosis as well as improved treatment outcomes, resulting in shorter hospitalization periods.

Yet, as TB patients are generally poor, higher hospitalization fees may create financial barriers to care. Revenues generated from in-patients through fee-for-service schemes outside the government-provided TB package of care may financially incentivize hospitals to unnecessarily retain patients through hospitalization [23]. High-costs associated with TB care may lead to interruption of treatment, including treatment failure, or the development of drug-resistant forms that are clinically more complicated and cost more to treat [2,15,24]. Health coverage and social protection mechanisms should be expanded to mitigate rising costs towards the elimination of economic barriers to care.

Second, China’s previous models of hospital-based treatment that existed in the 1990s are associated with poorer treatment outcomes [2,15]. While health system reforms have occurred over the past two decades, in concentrating TB diagnostic and treatment services in hospitals, China will need to simultaneously implement regulations and monitoring mechanisms in order to avoid increased patient expenditure, longer hospitalization periods, and the irrational use of drugs, all of which are associated with lowered treatment success [2,15].

Third, treatment in hospitals also increases the possibility for primary transmission of TB, and healthcare workers are particularly at risk of infection [23,25,26]. Further, the threat of primary transmission of drug-resistant strains in such congregate settings presents additional challenges to infrastructure and patient administration. Given reforms, infection control in hospitals and other health care settings should be strengthened to minimize primary transmission.

Forth, although the two-month initial intensive treatment period is important for clinical outcome of TB patients, this study revealed that the days of hospitalization showed the decreasing trend from 1999 to 2009. The large number of in-patients in recent years may be responsible for this trend. In addition, the limitation of the hospital stays from the health administration was another reason for the shortened in-patient days. Based on our observations, there is the urgent need to improve the investment on the TB hospital to increase in-patient beds. Furthermore, the clinicians should enroll the refractory TB cases rather than all TB cases as in-patients to solve this dilemma.

Finally, regional disparities should also be addressed. Though fees associated with hospitalization were lowest in the western region, such expenditures to patients represented the highest proportion of regional GDP per capita. In comparison to the eastern and central regions, provinces in the western region have higher rates of TB [4,27,28], fewer TB hospitals [5], and slower economic development [17]. As part of on-going health and broader reforms, the Chinese government should continue to increase public investment in the western region. For TB control, this should include improved infrastructure, strengthened local diagnostic and treatment capacity, and social protection schemes for patients that aim to counteract regional disparities.

There are several limitations to the study. First, we only considered hospital fees, and did not collect other data on medical expenditures, such as diagnostic, treatment, and other medical service fees, or other costs associated with TB care, such as follow-up clinical appointments and transportation costs. As such, our results might not reflect actual treatment conditions and costs for TB patients. Second, we excluded costs associated with hospitalization of MDR-TB patients, who generally spend two months during the intensive phase as in-patients. Additional studies could highlight such expenditure, particularly given that MDR-TB treatment in China is not currently fully covered by health insurance schemes. Third, several co-morbidities and demographic characteristics contribute to the total cost and duration of hospitalization, which should be under consideration in the future research.

In conclusion, our findings show that the total number of in-patients and hospitalization fees increased from 1999 to 2009, though fees as a proportion of GDP per capita decreased. The average length of hospitalization decreased over this period. The large number of in-patients in recent years may be responsible for this trend, and limited space may have further contributed to shortened length of hospitalization. Based on our observations, there is an urgent need to bolster investment in TB specialized hospitals, such as increasing the number of dedicated TB beds. Furthermore, clinicians should reserve hospital beds for refractory TB cases. As diagnostic services move to hospitals, regulatory and monitoring mechanisms should be put in place, and hospitals should make use of the experience garnered by the CDC system through stronger collaborations. Infrastructure and social protection mechanisms in high incidence settings, such as in the western region, should be strengthened.

## Supporting Information

S1 Table Number of TB in-patientsamong different regions of China. (DOCX)

S2 Table GDP per person in 1999, 2004 and 2009 among different regions of China. (DOCX)
