# Supplementary material for: Tuberculosis Hospitalization Fees and Bed Utilization in China from 1999 to 2009: The Results of a National Survey of Tuberculosis Specialized Hospitals
Source: PLoS One. 2015 Oct 12;10(10):e0139901. doi: 10.1371/journal.pone.0139901 (PMC4601762; doi:10.1371/journal.pone.0139901)
Supplement: S1 Table — (DOCX) [file pone.0139901.s001.docx]

| Year | No. of TB in-patients | | | |
| --- | --- | --- | --- | --- |
|  | Eastern | Central | Western | Total |
| 1999 | 43989 | 39659 | 9060 | 92708 |
| 2004 | 68067 | 64814 | 18898 | 151779 |
| 2009 | 115665 | 109737 | 39108 | 264510 |
