# Supplementary material for: Tuberculosis Hospitalization Fees and Bed Utilization in China from 1999 to 2009: The Results of a National Survey of Tuberculosis Specialized Hospitals
Source: PLoS One. 2015 Oct 12;10(10):e0139901. doi: 10.1371/journal.pone.0139901 (PMC4601762; doi:10.1371/journal.pone.0139901)
Supplement: S2 Table — (DOCX) [file pone.0139901.s002.docx]

| Year | GDP per capita (RMB) | | |
| --- | --- | --- | --- |
|  | Eastern | Central | Western |
| 1999 | 12700 | 5412 | 4632 |
| 2004 | 23130 | 9844 | 8462 |
| 2009 | 44732 | 21022 | 19306 |
